# Frequency of the common promoter polymorphism *MMP2 −1306 C>T* in a population from central Bulgaria

**DOI:** 10.1080/13102818.2014.995411

**Published:** 2015-01-28

**Authors:** Tanya Tacheva, Pavlina Chelenkova, Dimo Dimov, Rumena Petkova, Stoyan Chakarov, Tatyana Vlaykova

**Affiliations:** ^a^ Department of Chemistry and Biochemistry, Medical Faculty, Trakia University, Stara Zagora, Bulgaria; ^b^ STS Ltd., Sofia, Bulgaria; ^c^ Department of Internal Medicine, Medical Faculty, Trakia University, Stara Zagora, Bulgaria; ^d^ Department of Biochemistry, Faculty of Biology, Sofia University “St Kliment Ohridski”, Sofia, Bulgaria

**Keywords:** MMP2, SNP, MAF, genotyping

## Abstract

Matrix metalloproteinases (MMPs) are a family of highly homologous extracellular Zn^2+^-dependent endopeptidases, also known as matrixins. MMP-2 (gelatinase A) and MMP-9 (gelatinase B) are considered to play a key role in a variety of physiological processes as well as in the development and progression of a vast majority of pathological conditions. Most of the genes encoding MMPs, including MMP-2, are highly polymorphic. One of the single nucleotide polymorphisms with functional activity in the promoter region of *MMP2* is the transition *MMP2 −1306C>T* (rs243865). The aim of the present study was to evaluate the genotype and allele frequencies of the common promoter polymorphism *−1306C>T* in *MMP2* in 75 individuals from central Bulgaria and to compare our results with those of other population studies. We found that 76.0% of the randomly enrolled individuals are carriers of the *CC* genotype, 17.3% of *CT*, and 6.7% of the *TT* genotype. The minor allele frequency (MAF) was 15.3%. Interestingly, the obtained genotype frequencies appeared to differ from those of some other Caucasian populations (USA – 55/38/7, MAF 26%; The Netherlands – 52.8/40.5/6.7, MAF 26.9%; Austria – 55.6/35.5/8.9, MAF 27.2%), but were closer to the values of the reported global genotype distribution (75.3/21.3/3.4, MAF 14%).

## Introduction

Multicellular organisms require an appropriate assembly of the extracellular matrix (ECM), which is essential for organizing tissues and organs and for functions and communications between cells. Coordinated changes in ECM composition (breakdown, synthesis and remodelling) are crucial for a variety of normal biological processes such as embryonic development, organ morphogenesis and ovulation.[1–4] On the other hand, the abnormal degradation of ECM proteins, either enhanced or decreased, occurs in a large number of pathological processes, such as cancer invasion and metastasis, rheumatoid arthritis, osteoarthritis, gastric ulcer, corneal ulceration, liver cirrhosis, fibrotic lung disease, atherosclerosis and chronic lung diseases.[1–8] The degradation of basement membrane (BM) and ECM proteins is accomplished by several proteolytic enzymes, which are released by a variety of cells. According to the amino acid residue or cofactor required for their activity, proteolytic enzymes can be divided into the following four groups: serine proteinases (e.g. plasminogen activators, PAs), lysosomal aspartyl and cysteine proteinases (cathepsins) and metalloproteinases, particularly matrix metalloproteinases (MMPs).[5–7,9]

MMPs are a large family of structurally related Zn^2+^-dependent neutral endopeptidases, also known as matrixins. They are able to cleave virtually all protein components of the ECM and BM. Moreover, they can hydrolyse clotting factors, cell–cell and cell–matrix adhesion molecules, cell-membrane precursor forms of growth factors, growth-factor-binding proteins, growth factor receptors, other proteinases and proteinase inhibitors, as well as their own inactive zymogene forms.[7,10,11]

In humans, the family of MMPs consists of more than 20 members which differ in substrate specificity, regulation and interactions with other MMP family members and TIMPs (tissue inhibitors of metalloproteinases).[11–15] Depending on their substrate specificity, MMPs are classified into five main groups: collagenases (MMP-1, MMP-8 and MMP-13), stromelysins (MMP-3, MMP-10 and MMP-11), gelatinases (MMP-2 and MMP-9), matrilysins (MMP-7 and MMP-26) and membrane-type MMPs (MT-MMPs).[11–15] MMPs are active at physiological pH and are secreted as zymogens, which require extracellular activation.[10,11,14–16]

Gelatinases, MMP-2 (72 kDa type IV collagenase, gelatinase A) and MMP-9 (92 kDa type IV collagenase, gelatinase B), are principally involved in the degradation of denatured collagens (gelatine) and a broad spectrum of ECM molecules such as native types I, II, III, IV, V, VII, X and XI collagens, elastin, fibronectin, vitronectin, laminin, aggrecan, entactin and tenascin.[11,14] MMP-2 is also able to break down many non-ECM molecules, including pro-IL-1β, pro-TNF-α (pro-Tumor necrosis factor-α), latent TGF-β (transforming growth factor-β), pro-IL-8, MCP-3 (monocyte chemoattractant protein-3), α_2_-macroglobulin, IGFBP-3 and -5 (insulin-like growth factor binding protein-3 and -5) and FGFR1 (fibroblast growth factor receptor 1). MMP-2 can also cleave and activate several MMP zymogens as proMMP-1, proMMP-2, proMMP-9 and proMMP-13.[6,8,11,14]

The activity of MMPs is under strict control by regulation of gene transcription, latent zymogene activation, interaction with specific ECM components and inhibition by endogenous inhibitors.[10,11,14,15] The expression of MMPs is induced by cytokines, growth factors, chemical agents, tumour promoters, physical stress, oncogenic transformation, cell–matrix and cell–cell interactions.[10–12,14,17] Extracellular stimuli activate transcriptional factors that bind to specific DNA sequences in 5′-regulatory regions of genes. Furthermore, the response to those extracellular stimuli depends on the structure and function of tissue-specific regulatory elements of the *MMP* genes.[10,11,17]

It has been revealed that the *MMP2* promoter contains the sequences of a number of potential *cis*-acting regulatory elements for a variety of transcription factors, including YB-1, Sp1, AP-1, AP-2, Ets-1, Stat3, p53, C/EBR, CREB, PAE3 and FoxM1.[18–20] It is suggested that the Sp1-binding site determines the basal activity of the *MMP2* promoter.[18]

Most of the genes encoding MMPs, including *MMP2*, are highly polymorphic and possess sequence variations in their regulatory regions.[10,21] There is strong evidence that the expression level of *MMP2* is affected by polymorphisms in the promoter region of the gene. One of the single nucleotide polymorphisms with functional activity in the promoter region of *MMP2* is the transition *MMP2 −1306C>T* (rs243865).

To the best of our knowledge, there is no information so far about the allele and genotype profile of *MMP2 −1306 C>T* in any Bulgarian population. In this respect, the aim of the present study was to evaluate the genotype and allele frequencies of the common promoter polymorphism *−1306 C>T* (rs243865) in *MMP2* in a population from central Bulgaria. We also compared our results with population studies on other Caucasian populations and other ethnicities and races.

## Materials and methods

### Subjects

Seventy-one unrelated subjects of Caucasian origin from the area of Stara Zagora (Bulgaria) were included in this study. There were 38% (27/71) males and 62% (44/71) females, aged between 30 and 79 years with a median of 60.5 years (mean of 60.50 ± 10.79 years). Only three of the individuals were of Turkish ethnic origin, while the rest (68) were of Bulgarian ethnicity. All participants gave their informed consent.

### DNA isolation and genotyping

Genomic DNA was isolated from 0.2 mL of whole blood, using a commercial kit for isolation of genomic DNA from blood (GenElute™ Mammalian Genomic DNA Miniprep Kit, Sigma, USA).

The genotyping for *MMP2 −1306 C>T* (rs243865) was carried out by the method of polymerase chain reaction–restriction fragment length polymorphism (PCR-RFLP) as described earlier.[22] Each reaction with a total volume of 15 μL contained 1.5 μL of 10× PCR buffer (with 25 mmol/L MgCl_2_), 1.2 μL of 2.5 μmol/L of deoxyribonucleosides (dNTPs), 0.6 U of Taq DNA polymerase, 7.6 pmol of each primer and 100 ng of DNA. The sequences of the primers were as follows: *MMP2*F: 5′-CTT CCT AGG CTG GTC CTT ACT GA-3′; *MMP2*R: 5′-CTG AGA CCT GAA GAG CTA AAG AGC T-3′. The temperature profile of the PCR reactions included primary denaturing of template DNA for 5 min at 94 °C, followed by 30 cycles of denaturation for 30 s at 94 °C, annealing for 30 s at 64 °C and polymerization for 30 s at 72 °C. The PCR reaction was competed by a final extension for 1 min at 72 °C.

The restriction reaction of 4 μL of each PCR product mix was carried out with 5U *XspI* in a final volume of 15 μL for 16 h at 37 °C. The fragments obtained after the restriction reactions were analysed by 10% polyacrylamide gel electrophoresis (PAGE). PAGE was carried out in 1× ТВЕ (Tris–borate–ethylenediaminetraacetic acid) electrophoretic buffer and a field gradient of the electric current of 10–20 V/cm for 3.5 h.[23] The gels were silver stained and documented with a gel documentation system (Syngene, Synoptics Ltd., UK).

### Statistical analyses

Statistical analyses were performed using SPSS 16.0 (SPSS Inc.). *Chi*-square test was applied for comparing the obtained genotype and allele frequencies in our study with those reported by other authors.

## Results and discussion

### Frequency of *MMP2 −1306 C>T* genotypes in the studied population

**Figure 1.**
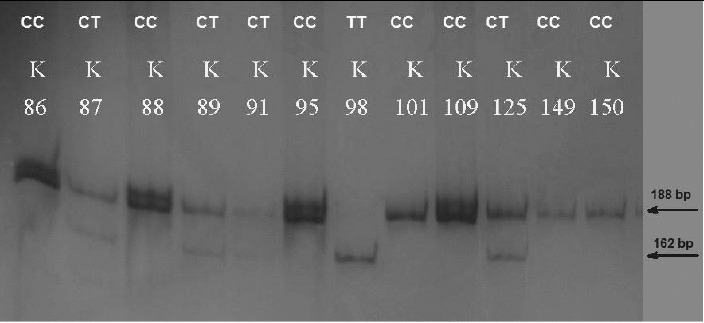
PAGE for visualization of PCR-RFLP products and genotyping for *MMP2* −*1306C>T*. The homozygous *CC* carriers were determined with one visible band of 188 bp (K86, K88, K95, K101, K109, K149, K150); the heterozygous *CT* carriers with two visible bands of 188 bp and 162 bp (K87, K89, K91, K125) and the homozygous *TT* carriers with one visible band of 162 bp (K98).

The PCR product amplified with the primers for *MMP2 −1306 C>T* SNP was 193 bp in length. The *XspI* digestion resulted in two fragments with a length of 188 and 5 bp for allele *C* and in three fragments with a length of 162, 26 and 5 bp for allele *T* (Figure 1).

**Figure 2.**
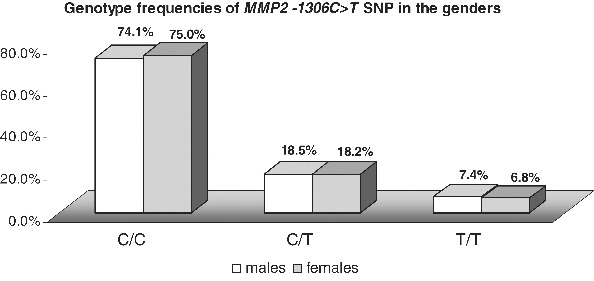
Comparison of the genotype and allele frequencies of *MMP2* −*1306C>T* SNP between genders.

In the whole studied population group, the homozygous *CC* carriers were 74.6% (53/71), *CT* carriers were 18.3% (13/71) and 7% were *TT* homozygous (5/71). The minor allele frequency (MAF) in the studied population was 16.2% (23/142). When only the individuals of Bulgarian ethnicity were analysed, the proportions of the genotypes were similar: 75.0% (51/68); 17.6% (12/68); 7.4% (5/68), MAF 16.2%. Moreover, there was no difference in the genotype frequencies between genders (*p* > 0.05) (Figure 2). All these findings encourage analyses for evaluation of the *MMP2 −1306 C>T* SNP as a risk factor for different diseases, such as cancers, chronic obstructive pulmonary disease (COPD), bronchial asthma, atherosclerosis and myocardial infarction, without stratification for gender and ethnicity.

When we compared the obtained genotype and allele frequencies in our population with those of some other Caucasian populations both from Europe and the USA, it appeared that they differed significantly (Table 1). There was also difference from the reported genotype frequency in a population from Turkey,[24] a country neighbouring Bulgaria (Table 1). However, the genotype frequencies in our study population were commensurate with the values of the reported global genotype distribution (75.3/21/3/3.4, MAF 14%, *p* > 0.05) [25] (Table 1).

**Table 1.** Allele and genotype frequencies of the *MMP2* −*1306C>T* SNP in the studied Caucasian population compared to other populations.

	Minor allele frequency (MAF)		Genotype number and frequencies			
Country/populations		*p*-value	C/C N (%)	C/T N (%)	T/T N (%)	*p*-value
Central Bulgaria/Caucasians (our study)	16.2		53 (74.6)	13 (18.3)	5 (7.0)	
Sweden/Caucasians [26]	26.2	0.021	109 (52.4)	89 (42.8)	10 (4.8)	0.002
Texas, USA/Caucasians [27]	24.2	0.043	312 (57.2)	202 (37.1)	31 (5.7)	0.012
The Netherlands/Caucasians [28]	26.9	0.007	609 (52.8)	466 (40.5)	77 (6.7)	0.001
China/Asians [29]	14.3	0.609	92 (73.0)	32 (25.5)	2 (1.6)	0.208
China/Asians [22]	11.8	0.132	487 (78.0)	3137 (21.0)	6 (1.0)	0.003
Thailand [13]	12.8	0.297	190 (76.0)	56 (22.4)	4 (1.6)	0.115
Austria/Caucasians [30]	26.6	0.011	138 (55.6)	88 (35.5)	22 (8.9)	0.021
Turkey/Caucasians [24]	9.2	0.026	142 (81.6)	32 (18.4)	0 (0)	0.010
Global genotype distribution [25]	14.0	0.813	(75.3)	(21.3)	(3.4)	0.686

### MMP2 promoter polymorphisms as risk factors

Gelatinase A (MMP-2) and gelatinase B (MMP-9) have three repeats of a type II fibronectin domain inserted in the catalytic domain, which bind to gelatin, collagens and laminin.[14] MMP-2 digests collagen I, IV, V, VII, X, XI and XIV, gelatin, elastin, fibronectin, laminin, and a proteoglycan-associated protein, osteonectin. MMP-9 digests collagen IV, V, VII, X, XIV, gelatin, elastin, aggrecan, proteoglycan-associated protein, fibronectin, osteonectin and plasminogen.[8]

MMP-2 (gelatinase A) is primarily expressed in mesenchymal cells (mainly fibroblasts) during development and tissue regeneration.[31,32] It is also synthesized by neutrophils, macrophages and monocytes.[14,31,32] MMP-2 is required to activate angiogenesis in tumours, and its level is increased in the endothelium of tumour vessels and in the urine of patients with different tumour entities.[8] Under normal physiological conditions, the activities of MMPs are subject to strict regulation at multiple levels: transcription, activation of their precursor zymogens, interaction with specific components of the ECM and inhibition by endogenous inhibitors.[7,8,33]

Most of the genes encoding MMPs are highly polymorphic. A variety of single-nucleotide polymorphisms (SNPs) have been found in the promoter regions of *MMP* genes.[2,7,14,34] These polymorphisms are associated with altered gene expression and enzyme activity of MMPs, which might eventually affect the individual susceptibility to different diseases.

So far, there are several known functionally active SNPs within the promoter region of *MMP2*: *−1575G>A*, *−1306 C>T* (rs243865), *−1015C>T* (rs2285053, also known as *−735C>T*) and *−168G>T*.[3,35–39] Two of the promoter SNPs with functional activity are the transitions *MMP2 −1306 C>T* (rs243865) and *−735C>T*. Both of them have been found to influence the Sp1-binding site in *MMP2* and to alter the transcription activity of the promoter.[35,36] *MMP2 −1306 C>T* is located at a core recognition sequence of Sp1 (CCACC box), resulting in strikingly lower promoter activity of the *T* allele due to loss of an Sp1-binding site in the promoter.[35] The other C→T transition located at nucleotide *−*735 in the promoter region of *MMP2* has also been found to destroy an Sp1-binding element, with the *T* allele being associated with significantly diminished promoter activity.[36,40] Moreover, both SNPs appear to be in linkage disequilibrium and the *T_−1306_T_−735_* haplotype has been shown to display a 7-fold lower luciferase expression and 3.7-fold decreased *MMP2* mRNA levels in oesophageal tissues compared with the *C_−1306_C_−735_* haplotype.[36] The *T_−1306_T_−735_* haplotype had even lower promoter activity and mRNA expression than the haplotypes consisting of only one T allele either at *−*1360 or *−*735 site. These data clearly indicate that there is an interactive effect of these two SNPs on the transcriptional function of the *MMP2* promoter.[36,40]

Several case–control studies aiming to elucidate the effect of these polymorphisms as risk factors for a variety of diseases have found that the allele and genotype frequencies of *MMP2 −1306 C>T* vary significantly between races and ethnicities: the variant *T* allele appears to be more common among Caucasians from northern European countries and the USA, but is less often determined in Caucasians from Turkey and in Asians.[13,22,24,26–30] So far, to the best of our knowledge, there are no reports concerning the allele and genotype profile of *MMP2 −1306 C>T* in any Bulgarian population.

Thus, our present study appears to be the first one describing the genotype profile of *MMP2 −1306C>T* SNP in Bulgarians. This study is a part of a larger project aiming to determine the genotype profile for different *MMP* gene variants in a Bulgarian Caucasian population from the central region of the country. Previously, we also described the genotype and allele distributions for two other functional SNPs (insertion/deletion) in *MMP* genes encoding MMP-1 (interstitial collagenase-1, *MMP1 −*1607insG, 1G/2G, rs1799750) and MMP-3 (stromelysin-1, *MMP3 −*1171insA, 5A/6A, rs3025058) [34] in Bulgarians. In that study, we did not find significant differences between the allele and genotype frequencies for promoter *MMP1* and *MMP3* polymorphisms in the population of Bulgarian Caucasians and most of the other Caucasian populations. Meanwhile, the observed frequencies were markedly distinguished from those reported in Asian populations.[34] Thus, based on the obtained remarkable similarities in the figures for other Caucasian-type populations, we concluded that Bulgarians do not differ from other Caucasians in the frequencies of *MMP1 −*1607insG and *MMP3 −*1171insA genotypes and could be included in larger inter-institutional case–control studies for investigation of the effect of this polymorphism on the susceptibility to different diseases, including cancers, COPD and bronchial asthma.

However, in the present study, the distributions of the genotypes for *MMP2 −1306 C>T SNP* deviated from those reported for other European Caucasian populations. The main limitation of our study is the relatively small number of individuals included. Another limitation is the very small number or absence of individuals of other than Bulgarian ethnic origin, such as Turkish, Roma and Armenian. In this respect, our data present only preliminary results and should not be considered as representative for the whole Bulgarian population.

## Conclusions

The present preliminary study on the *MMP2 −1306C>T* genotype profile in Bulgarians showed that 76.0% of the individuals were carriers of the *CC* genotype, 17.3% of *CT* and 6.7% of the *TT* genotype. This genotype distribution appeared to differ from those of some other Caucasian populations, but was commensurable to the values of the reported global genotype distribution (75.3/21.3/3.4, MAF 14%). In this respect, further analyses with a larger population group consisting of individuals from different ethnicities and regions of Bulgaria are required, which will be our task in our future work.

## Disclosure statement

No potential conflict of interest was reported by the authors.
